# Supplementary material for: Price volatility and GHG emissions analysis on smaller cattle herds typical for the pre-Alpine region, the example of Slovenia
Source: PLoS One. 2024 Nov 22;19(11):e0310713. doi: 10.1371/journal.pone.0310713 (PMC11584099; doi:10.1371/journal.pone.0310713)
Supplement: Attachment — Submitted filename: Response to reviewers.docx [file pone.0310713.s002.docx]

1. Please ensure that your manuscript meets PLOS ONE's style requirements, including those for file naming. The PLOS ONE style templates can be found at 
<https://journals.plos.org/plosone/s/file?id=wjVg/PLOSOne_formatting_sample_main_body.pdf> and 
<https://journals.plos.org/plosone/s/file?id=ba62/PLOSOne_formatting_sample_title_authors_affiliations.pdf>

The manuscript has been checked and ensured to meet PLOS ONE style requirements, including formatting of headings, using double spaced paragraph format and sorting of references.

2. Please note that PLOS ONE has specific guidelines on code sharing for submissions in which author-generated code underpins the findings in the manuscript. In these cases, all author-generated code must be made available without restrictions upon publication of the work. Please review our guidelines at <https://journals.plos.org/plosone/s/materials-and-software-sharing#loc-sharing-code> and ensure that your code is shared in a way that follows best practice and facilitates reproducibility and reuse.

The raw data supporting the conclusions of this article will be made available by the authors on request.«

3. We note that the grant information you provided in the ‘Funding Information’ and ‘Financial Disclosure’ sections do not match.

When you resubmit, please ensure that you provide the correct grant numbers for the awards you received for your study in the ‘Funding Information’ section.

4. Thank you for stating the following financial disclosure: 
"The authors acknowledge core financing grants (P4-0133 and P4-0022) of the Slovenian 
Research and Innovation Agency."

Please state what role the funders took in the study.  If the funders had no role, please state: **""The funders had no role in study design, data collection and analysis, decision to publish, or preparation of the manuscript**."" 
If this statement is not correct you must amend it as needed. 
Please include this amended Role of Funder statement in your cover letter; we will change the online submission form on your behalf.

The funders had no role in study design, data collection and analysis, decision to publish, or preparation of the manuscript.

5. Thank you for stating the following in the Acknowledgments Section of your manuscript: 
"The authors acknowledge core financing grants (P4-0133 and P4-0022) of the Slovenian 
Research and Innovation Agency."

We note that you have provided funding information that is not currently declared in your Funding Statement. However, funding information should not appear in the Acknowledgments section or other areas of your manuscript. We will only publish funding information present in the Funding Statement section of the online submission form. 
Please remove any funding-related text from the manuscript and let us know how you would like to update your Funding Statement. Currently, your Funding Statement reads as follows: 
"The author(s) received no specific funding for this work"

Please include your amended statements within your cover letter; we will change the online submission form on your behalf.

The authors acknowledge core financing grants (P4-0133 and P4-0022) of the Slovenian Research and Innovation Agency (ARIS).

6. Please amend the manuscript submission data (via Edit Submission) to include author Dr. Jaka Žgajnar
and Dr. Črtomir Rozman.

The manuscript submission data has been updated.

7. Please ensure that you refer to Figure 1 and 2 in your text as, if accepted, production will need this reference to link the reader to the figure.

This has been corrected as both figures are now included in the text.

8. Please review your reference list to ensure that it is complete and correct. If you have cited papers that have been retracted, please include the rationale for doing so in the manuscript text, or remove these references and replace them with relevant current references. Any changes to the reference list should be mentioned in the rebuttal letter that accompanies your revised manuscript. If you need to cite a retracted article, indicate the article’s retracted status in the References list and also include a citation and full reference for the retraction notice.

A few references have been added to the manuscript list and the citation of references has been updated to the demands of PLOS ONE guidelines. Added citations to the manuscript are:

- Eliasson Å, Jones RJA, Nachtergaele F, Rossiter DG, Terres JM, Van Orshoven J, et al. Common criteria for the redefinition of Intermediate Less Favoured Areas in the European Union Environ. Sci. Policy. 2010; 13: 766-777
- Bedrač M, Bele S, Bleiweis A, Brečko J, Kožar M, Moljk B, et al. Green report for year 2022. Travnikar T (E.) Report on the state of agriculture, food, forestry and fisheries in 2022. Ljubljana, Agricultural Institute of Slovenia. 2023; [in Slovene]
- Pastusiak R, Soliwoda M, Jasiniak M, Stawska J, Pawłowska-Tyszko J. Are Farms Located in Less-Favoured Areas Financially Sustainable? Empirical Evidence from Polish Farm Households. Sustainability. 2021; 13(3): 1092
- Barnes AP. The role of family life-cycle events on persistent and transient inefficiencies in less favoured areas. J. Agric. Econ. 2023; 74 (1): 295-315
- Louhichi K, Ciaian P, Espinosa M, Colen L, Perni A, Gomez y Paloma S. An EU‐Wide Individual Farm Model for Common Agricultural Policy Analysis (IFM‐CAP). JRC Scientific and policy reports. 2015
- van der Linden A, de Olde EM, Mostert PF, de Boer IJM. A review of European models to assess the sustainability performance of livestock production systems. Agric. Syst. 2020; 182

Reviewer's Responses to Questions

Thank you for the observations and comments.

**Comments to the Author**

1. Is the manuscript technically sound, and do the data support the conclusions?

The manuscript must describe a technically sound piece of scientific research with data that supports the conclusions. Experiments must have been conducted rigorously, with appropriate controls, replication, and sample sizes. The conclusions must be drawn appropriately based on the data presented.

Reviewer #1: Yes

2. Has the statistical analysis been performed appropriately and rigorously?

Reviewer #1: Yes

3. Have the authors made all data underlying the findings in their manuscript fully available?

The [PLOS Data policy](http://www.plosone.org/static/policies.action#sharing) requires authors to make all data underlying the findings described in their manuscript fully available without restriction, with rare exception (please refer to the Data Availability Statement in the manuscript PDF file). The data should be provided as part of the manuscript or its supporting information, or deposited to a public repository. For example, in addition to summary statistics, the data points behind means, medians and variance measures should be available. If there are restrictions on publicly sharing data—e.g. participant privacy or use of data from a third party—those must be specified.

Reviewer #1: Yes

4. Is the manuscript presented in an intelligible fashion and written in standard English?

PLOS ONE does not copyedit accepted manuscripts, so the language in submitted articles must be clear, correct, and unambiguous. Any typographical or grammatical errors should be corrected at revision, so please note any specific errors here.

Reviewer #1: No

Thank you for the comment. The manuscript has gone through a lecture and has been properly corrected in its entirety.

5. Review Comments to the Author

Please use the space provided to explain your answers to the questions above. You may also include additional comments for the author, including concerns about dual publication, research ethics, or publication ethics. (Please upload your review as an attachment if it exceeds 20,000 characters)

Reviewer #1: The authors attempt to formulate microsimulation tools to analyse how specific conditions affect farm operations and address different technological challenges at farm and technology sector level. This paper presents a farm model for the analysis of the cattle sector in the pre-alpine region in the context of economic results and ecological effects.
The aim of work is to examine the impact of price volatility (input and output prices) on economic results at the farm and sector level in the period 2018-2022. It was characterised by pronounced upheavals while simultaneously analysing GHG emissions using a farm model. The paper presents (i) an analysis of the impact of price volatility on the economic performance of the cattle sector on typical Slovenian farms and (ii) a parallel estimation of the greenhouse gas emissions of the cattle sector on this type of farm (smaller size).
The authors believe that the methodology used has proved successful as it allows simulations to be carried out at both farm and cattle sector level and can be applied to the analysis of the cattle sector in the pre-Alpine region. I would be more cautious in this type of conclusion. The period analysed is relatively short (5 years), the external conditions (economic, political, environmental) very unstable, the case study concerns the behaviour of small farms, which are the most difficult to predict and very diverse in terms of operating conditions.

Thank you for the comment. We have revised the conclusion a little bit, to add the remark of analysis carried out in relatively short period, with unstable external conditions. Our model allows for a simulation of the defined period and long term predictions based on these results are not viable.

In principle, I have no objections to the econometric side of the study (data to be analysed and model), but the theoretical introduction and analysis of the literature on the subject (small livestock farms in the mountain regions of Europe) is far too modest.

Thank you for your observation. We have added a paragraph in the introduction referencing farms in LFA (Less-Favoured Areas). In this section, we emphasized that LFA areas include not only mountainous regions but also naturally handicapped areas such as those with challenging climatic conditions, steep slopes, or low soil productivity. Our analysis is intended to cover all LFA farms in the region, not just those in mountainous areas. We highlighted that these are small livestock farms compared to major EU producers, yet they play a crucial role in environmental and social sustainability. We hope these revisions meet your expectations.

I am also not sure that combining two issues in one model: the economic viability of livestock farms in the mountains and their environmental impact is not too much to ask. There are few specific statements in the conclusions relating to this interaction.

Thank you for your comment. Our research is not intended to combine the two issues but rather to complement traditional economic viability studies with an environmental component, which is increasingly important in the context of climate change. This addition provides an additional layer of information for decision-makers. We also addressed this aspect in the conclusion, highlighting that the results can be valuable for decision-makers and agricultural policy planners.

As an aside: I think there is a mistake in the title: I suppose it is about the Pre-Alpine region, not the Pre-Alpal region (I have not come across this type of term in the geographical literature).

Thank you for the comment. It has been corrected throughout the paper.

6. PLOS authors have the option to publish the peer review history of their article ([what does this mean?](https://journals.plos.org/plosone/s/editorial-and-peer-review-process#loc-peer-review-history)). If published, this will include your full peer review and any attached files.


If you choose “no”, your identity will remain anonymous but your review may still be made public.


**Do you want your identity to be public for this peer review?** For information about this choice, including consent withdrawal, please see our [Privacy Policy](https://www.plos.org/privacy-policy).

Reviewer #1: **Yes:** Krystian Heffner, University of Economics in Katowice
